# Who can do without patients?

**DOI:** 10.1007/s40037-015-0171-2

**Published:** 2015-03-31

**Authors:** Olle ten Cate, Max Peters

**Affiliations:** Center for Research and Development of Education, University Medical Center Utrecht, PO Box 85500, 3508 GA Utrecht, The Netherlands

We appreciate Cantillon and Dornan’s comment [1] on our observation [2] that in the medical education literature and textbooks bedside teaching is gradually disappearing. The authors make the case that *beds* are less functional in medical education and that *teaching* is not the most appropriate word for the role of clinical educators, and therefore our concern may be less alarming.

Linguistically, this may be true, but the interpretation of our message is narrower and more literal than it was meant. The deeper point of our regret is that the patient seems to become less central in workplace education. The loss of instructional texts about how to role model in the presence of patients in the sense of bedside teaching may be illustrative of the decreased educational time spent at the side of the patient in general, including in ambulatory care. Doctors and medical trainees now spend less and less time with patients [3] than in the past. A recent study found that interns spend 12 % on average in direct patient care and 40 % behind a computer screen. The reasons for this lie in restricted working hours, restricted time for patients in hospital settings, short rotations and an increased volume of patient data to be handled. It may to some extent be true that patients are less in bed, but predominantly students do not *see* them in bed. In addition, students and residents are hardly observed when working with patients and hardly observe clinicians doing this [4, 5].

Why is that not good? A lack of patient-related education is detrimental for the development of clinical thinking and reasoning skills, and for professional identity formation and professional behaviour. Clinical diagnostic decision-making is primarily based on pattern recognition [6], which requires extensive experience with patients. The more the better. Many valid illness scripts in the long-term memory of clinicians are needed to adequately recognize patterns or stimulate analytic reasoning. It has long been recognized that an adequate history is associated with better diagnosis and management than tests [7]. In education, seeing patients is one, but an experienced educator and role model is needed to help turn this seeing into deliberate practice [8]. Longitudinal clerkships may restore continuity in both patient care and clinical teaching [9] if we may use this word teaching to signify the variety of activities of committed clinician educators, including role modelling, coaching, stimulating reflection and allocating learning opportunities to match the student’s developmental level [10].

Elder et al. recently mapped a road back to the bedside [11], or should we say, back to having clinical teachers, learners and patients together discuss medicine?
